# Supplementary material for: Targeted Analysis of Sphingolipids in Turkeys Fed Fusariotoxins: First Evidence of Key Changes That Could Help Explain Their Relative Resistance to Fumonisin Toxicity
Source: Int J Mol Sci. 2022 Feb 24;23(5):2512. doi: 10.3390/ijms23052512 (PMC8910753; doi:10.3390/ijms23052512)
Supplement: Supplementary file 1 [file ijms-23-02512-s001.zip › ijms-1593023-supplementary.pdf]

**Table S1.** Linearity of the method measured for the 33 sphingolipids available as standards.

| Analyte[1] | Conc[2] | Eq. Conc[3] | a[4] | b[4] | $(R^2)^4$ | RSD (%)[5] |
|---|---|---|---|---|---|---|
| dSo | 0.1-6 | 2-120 | 0.9955 | 0.0124 | 0.9999 | 8 |
| dSa | 0.1-6 | 2-120 | 0.9932 | 0.0185 | 0.9997 | 3 |
| d18:1 (So) | 16-4000 | 320-80,000 | 0.9985 | 2.1619 | 1 | 4 |
| d18:0 (Sa) | 8-2000 | 160-40,000 | 0.9971 | 1.5219 | 1 | 3 |
| 18:1/2:0 | 1-63 | 20-1260 | 1.0077 | 0.1517 | 0.9999 | 7 |
| 18:0/2:0 | 2-125 | 40-2500 | 0.9898 | 0.6935 | 0.9996 | 7 |
| d18:1P | 63-1000 | 1260-20,000 | 1.0021 | -2.1379 | 0.9989 | 7 |
| d18:0P | 63-1000 | 1260-20,000 | 1.0339 | -28.614 | 0.9989 | 12 |
| GluSo | 0.5-31 | 10-620 | 0.9857 | 0.1586 | 0.9993 | 6 |
| LysoSM | 2-31 | 40-620 | 1.0011 | 0.0265 | 1 | 10 |
| 18:1/14:0 | 4-250 | 80-5000 | 1.0012 | -0.2492 | 1 | 6 |
| 18:1/16:0 | 500-32,000 | 10,000-640,000 | 0.9969 | 31.042 | 1 | 8 |
| 18:0/16:0 | 16-4000 | 320-80,000 | 0.9977 | 2.5003 | 1 | 11 |
| 18:1/18:0 | 63-4000 | 1260-80,000 | 1.0017 | -3.1509 | 1 | 13 |
| 18:1/20:0 | 31-2000 | 620-40,000 | 1.0049 | -4.2797 | 0.9999 | 12 |
| 18:1/16:0P | 63-1000 | 1260-20,000 | 1.0008 | -1.0981 | 1 | 17 |
| 18:1/22:0 | 250-16,000 | 5000-320,000 | 0.9856 | 65.638 | 0.9996 | 9 |
| LacSo | 2-125 | 40-2500 | 0.8262 | 2.1642 | 0.9978 | 10 |
| 18:1/24:1 | 250-16,000 | 5000-320,000 | 0.9963 | 50.453 | 0.9999 | 7 |
| 18:1/24:0 | 125-8000 | 2500-160,000 | 0.9985 | 4.2147 | 1 | 14 |
| 18:0/24:0 | 8-2000 | 160-40,000 | 1.122 | -25.72 | 0.9994 | 17 |
| SM18:1/14:0 | 2-500 | 40-20,000 | 1.1542 | -5.5697 | 0.9989 | 5 |
| Glu18:1/16:0 | 16-4000 | 320-80,000 | 1.004 | -5.5704 | 0.9999 | 13 |
| SM18:1/16:0 | 250-16,000 | 5000-320,000 | 1.0082 | -42.218 | 1 | 5 |
| SM18:1/18:1 | 4-1000 | 80-20,000 | 1.0771 | -5.5818 | 0.9997 | 4 |
| SM18:1/18:0 | 125-32,000 | 5000-320,000 | 1.0055 | -21.454 | 1 | 9 |
| SM18:1/20:0 | 16-4000 | 320-80,000 | 0.9999 | 0.4259 | 1 | 10 |
| SM18:1/22:0 | 250-16,000 | 5000-320,000 | 1.0061 | -40.417 | 1 | 13 |
| Glu18:1/24:1 | 8-2000 | 160-40,000 | 1.0008 | -07942 | 1 | 12 |
| SM18:1/24:1 | 125-32,000 | 5000-640,000 | 1.0575 | -178.12 | 0.9995 | 9 |
| SM18:1/24:0 | 16-4000 | 320-80,000 | 0.999 | 1.5599 | 1 | 11 |
| Lac18:1/16:0 | 16-4000 | 320-80,000 | 1.0017 | -3.0115 | 1 | 15 |
| Lac18:1/24:1 | 8-2000 | 160-40,000 | 1.0898 | -18.404 | 0.9989 | 13 |

[1]dSo= deoxy-sphingosine, dSa= deoxy-sphinganine, So = sphingosine, Sa = sphinganine, GluSo = glucosyl-sphingosine, LysoSM = lyso-sphingomyelin, LacSo = Lactosylsphingosine, SM = sphingomyelin, Glu = glucosyl, Lac = lactosyl

[2]Conc = concentration in the net solvent expressed in pmol/mL, ratio between each concentration tested is 4, n = 4 per concentration

[3]Eq. Conc = equivalent concentration in liver expressed in pmol/g

[4]calibration curve: a = slope, b = y-intercept, $R^2$ = coefficient of determination

[5]RSD = relative standard deviation

**Table S2.** Intra-day and inter-day recovery of the internal standards measured in the liver of turkeys.

| | Intra-day[1] | | Inter-day[2] | |
|---|---|---|---|---|
| Analyte | R (%) | RSD (%) | R (%) | RSD (%) |
| d17:1 | 57 | 20 | 61 | 21 |
| d17:0 | 82 | 6 | 87 | 4 |
| d17:1P | 123 | 8 | 127 | 11 |
| d17:0P | 121 | 8 | 125 | 5 |
| 18:1/12:0 | 100 | 4 | 103 | 5 |
| 18:1/12:0P | 126 | 7 | 119 | 9 |
| Glud18:1C12:0 | 109 | 5 | 115 | 7 |
| SM18:1/12:0 | 96 | 6 | 98 | 3 |
| 18:1/25:0 | 78 | 25 | 80 | 20 |
| Lac18:1/12:0 | 117 | 6 | 122 | 6 |

[1]Intra-day recovery (R) and intra-day repeatability (RSD) measured the same day on 12 samples spiked with the internal standards before extraction at 6,250 pmol/g liver

[2]Inter-day recovery (R) and intra-day repeatability (RSD) measured on 3 consecutive days on 4 samples per day spiked with the internal standards before extraction at 6,250 pmol/g liver

**Table S3.** Spearman correlation measured among different sphingolipids *.

| Variables | dSa | So | Sa | 18:1/2:0 | 18:0/2:0 | GluSo | LysoSM | 18:1/14:0 | 18:1/16:0 | 18:0/16:0 | 18:1/18:0 | 18:1/20:0 | 18:1/22:0 | 18:1/24:1 | 18:1/24:0 | 18:0/24:0 | SM18:1/14:0 | Glc18:1/16:0 | SM18:1/16:0 | SM18:1/18:0 | SM18:1/20:0 | SM18:1/22:0 | Glu18:1/24:1 | SM18:1/24:1 | SM18:1/24:0 | Lct18:1/16:0 | 18:1/18:1 | 18:0/18:0 | 18:0/20:0 | 18:1/22:2 | 18:1/22:1 | 18:0/22:0 |
|---|---|---|---|---|---|---|---|---|---|---|---|---|---|---|---|---|---|---|---|---|---|---|---|---|---|---|---|---|---|---|---|---|
| dSa | **1** | **0.568** | **0.463** | **0.700** | **0.467** | 0.045 | 0.078 | **0.387** | **0.372** | **0.481** | **0.518** | 0.205 | **0.388** | **0.390** | **0.580** | **0.487** | **0.487** | 0.021 | **0.473** | **0.468** | **0.429** | **0.489** | **0.316** | **0.571** | **0.497** | 0.116 | **0.490** | **0.553** | **0.403** | 0.233 | **0.376** | **0.475** |
| So | **0.568** | **1** | 0.252 | **0.620** | 0.268 | 0.108 | 0.138 | 0.221 | **0.367** | **0.428** | **0.443** | **0.318** | **0.403** | **0.396** | **0.478** | **0.324** | 0.040 | 0.106 | 0.132 | 0.198 | 0.176 | 0.166 | **0.381** | 0.167 | 0.138 | 0.068 | **0.701** | **0.467** | **0.517** | **0.391** | **0.384** | **0.385** |
| Sa | **0.463** | 0.252 | **1** | **0.581** | **0.872** | 0.042 | 0.170 | **-0.376** | **-0.436** | **0.484** | 0.273 | 0.109 | **0.369** | 0.271 | **0.576** | **0.839** | 0.037 | **-0.536** | -0.161 | **0.429** | **0.536** | **0.712** | **0.339** | **0.541** | **0.744** | -0.221 | **0.518** | **0.764** | **0.378** | 0.182 | 0.155 | **0.781** |
| 18:1/2:0 | **0.700** | **0.620** | **0.581** | **1** | **0.540** | 0.155 | 0.062 | 0.128 | 0.192 | **0.471** | **0.721** | **0.388** | **0.576** | **0.443** | **0.708** | **0.565** | 0.279 | -0.093 | 0.282 | **0.621** | **0.588** | **0.604** | **0.407** | **0.526** | **0.558** | -0.047 | **0.711** | **0.726** | **0.424** | **0.387** | **0.453** | **0.580** |
| 18:0/2:0 | **0.467** | 0.268 | **0.872** | **0.540** | **1** | -0.037 | 0.108 | **-0.334** | **-0.389** | **0.512** | 0.236 | 0.186 | **0.407** | **0.397** | **0.504** | **0.763** | 0.053 | **-0.431** | -0.115 | **0.399** | **0.511** | **0.648** | **0.432** | **0.533** | **0.629** | -0.198 | **0.435** | **0.726** | **0.478** | **0.349** | **0.293** | **0.793** |
| GluSo | 0.045 | 0.108 | 0.042 | 0.155 | -0.037 | **1** | **0.759** | 0.000 | 0.140 | -0.237 | 0.113 | 0.175 | 0.193 | 0.169 | 0.209 | -0.104 | 0.166 | -0.057 | 0.190 | 0.162 | 0.105 | 0.072 | 0.008 | 0.062 | 0.065 | -0.128 | 0.125 | -0.089 | -0.116 | -0.040 | 0.154 | -0.069 |
| LysoSM | 0.078 | 0.138 | 0.170 | 0.062 | 0.108 | **0.759** | **1** | -0.105 | -0.035 | -0.175 | -0.078 | -0.025 | 0.034 | 0.080 | 0.159 | 0.068 | 0.155 | -0.062 | 0.101 | 0.075 | 0.086 | 0.088 | 0.112 | 0.132 | 0.141 | -0.037 | 0.144 | -0.014 | -0.045 | -0.155 | -0.014 | 0.032 |
| 18:1/14:0 | **0.387** | 0.221 | **-0.376** | 0.128 | **-0.334** | 0.000 | -0.105 | **1** | **0.827** | 0.234 | **0.315** | 0.076 | -0.021 | 0.072 | 0.011 | **-0.289** | **0.490** | **0.695** | **0.642** | 0.064 | -0.046 | -0.113 | 0.119 | 0.131 | -0.085 | **0.659** | -0.001 | -0.096 | -0.011 | 0.138 | 0.238 | **-0.295** |
| 18:1/16:0 | **0.372** | **0.367** | **-0.436** | 0.192 | **-0.389** | 0.140 | -0.035 | **0.827** | **1** | 0.172 | **0.353** | 0.145 | 0.065 | 0.168 | 0.063 | **-0.365** | **0.316** | **0.677** | **0.580** | -0.034 | -0.178 | -0.266 | 0.167 | -0.011 | -0.279 | **0.445** | 0.085 | -0.131 | 0.044 | 0.156 | 0.247 | **-0.311** |
| 18:0/16:0 | **0.481** | **0.428** | **0.484** | **0.471** | **0.512** | -0.237 | -0.175 | 0.234 | 0.172 | **1** | **0.417** | 0.052 | 0.226 | 0.226 | **0.407** | **0.608** | 0.038 | 0.224 | 0.120 | 0.191 | **0.295** | **0.408** | **0.684** | **0.378** | **0.430** | **0.405** | **0.516** | **0.742** | **0.480** | **0.431** | 0.199 | **0.565** |
| 18:1/18:0 | **0.518** | **0.443** | 0.273 | **0.721** | 0.236 | 0.113 | -0.078 | **0.315** | **0.353** | **0.417** | **1** | **0.526** | **0.655** | **0.428** | **0.705** | **0.449** | 0.140 | -0.042 | 0.252 | **0.704** | **0.637** | **0.566** | 0.224 | **0.355** | **0.483** | -0.035 | **0.664** | **0.679** | **0.389** | **0.317** | **0.514** | **0.465** |
| 18:1/20:0 | 0.205 | **0.318** | 0.109 | **0.388** | 0.186 | 0.175 | -0.025 | 0.076 | 0.145 | 0.052 | **0.526** | **1** | **0.913** | **0.854** | **0.647** | 0.200 | 0.069 | -0.209 | 0.074 | **0.432** | **0.339** | 0.229 | 0.016 | 0.013 | 0.061 | **-0.316** | **0.287** | **0.289** | **0.695** | **0.690** | **0.934** | **0.464** |
| 18:1/22:0 | **0.388** | **0.403** | **0.369** | **0.576** | **0.407** | 0.193 | 0.034 | -0.021 | 0.065 | 0.226 | **0.655** | **0.913** | **1** | **0.848** | **0.869** | **0.477** | 0.027 | **-0.359** | 0.010 | **0.528** | **0.478** | **0.455** | 0.124 | 0.167 | **0.302** | **-0.371** | **0.494** | **0.556** | **0.760** | **0.617** | **0.850** | **0.705** |
| 18:1/24:1 | **0.390** | **0.396** | 0.271 | **0.443** | **0.397** | 0.169 | 0.080 | 0.072 | 0.168 | 0.226 | **0.428** | **0.854** | **0.848** | **1** | **0.720** | **0.368** | 0.194 | -0.163 | 0.198 | **0.412** | **0.351** | **0.307** | 0.268 | 0.234 | 0.166 | -0.261 | **0.300** | **0.385** | **0.767** | **0.760** | **0.901** | **0.603** |
| 18:1/24:0 | **0.580** | **0.478** | **0.576** | **0.708** | **0.504** | 0.209 | 0.159 | 0.011 | 0.063 | **0.407** | **0.705** | **0.647** | **0.869** | **0.720** | **1** | **0.722** | 0.102 | **-0.344** | 0.068 | **0.578** | **0.562** | **0.620** | 0.280 | **0.387** | **0.556** | -0.212 | **0.651** | **0.742** | **0.704** | **0.414** | **0.628** | **0.804** |
| 18:0/24:0 | **0.487** | **0.324** | **0.839** | **0.565** | **0.763** | -0.104 | 0.068 | **-0.289** | **-0.365** | **0.608** | **0.449** | 0.200 | **0.477** | **0.368** | **0.722** | **1** | -0.009 | **-0.494** | -0.164 | **0.483** | **0.578** | **0.721** | **0.354** | **0.497** | **0.734** | -0.147 | **0.601** | **0.902** | **0.580** | 0.190 | 0.236 | **0.920** |
| SM18:1/14:0 | **0.487** | 0.040 | 0.037 | 0.279 | 0.053 | 0.166 | 0.155 | **0.490** | **0.316** | 0.038 | 0.140 | 0.069 | 0.027 | 0.194 | 0.102 | -0.009 | **1** | 0.252 | **0.895** | **0.511** | **0.407** | **0.364** | 0.081 | **0.706** | **0.370** | 0.266 | -0.201 | 0.002 | -0.047 | 0.109 | 0.258 | -0.078 |
| Glc18:1/16:0 | 0.021 | 0.106 | **-0.536** | -0.093 | **-0.431** | -0.057 | -0.062 | **0.695** | **0.677** | 0.224 | -0.042 | -0.209 | **-0.359** | -0.163 | **-0.344** | **-0.494** | 0.252 | **1** | **0.511** | -0.256 | **-0.303** | **-0.387** | **0.438** | -0.033 | **-0.327** | **0.787** | -0.139 | **-0.302** | -0.178 | 0.125 | -0.079 | **-0.504** |
| SM18:1/16:0 | **0.473** | 0.132 | -0.161 | 0.282 | -0.115 | 0.190 | 0.101 | **0.642** | **0.580** | 0.120 | 0.252 | 0.074 | 0.010 | 0.198 | 0.068 | -0.164 | **0.895** | **0.511** | **1** | **0.462** | **0.341** | 0.240 | 0.199 | **0.608** | 0.227 | **0.388** | -0.156 | -0.056 | -0.070 | 0.197 | 0.261 | -0.209 |
| SM18:1/18:0 | **0.468** | 0.198 | **0.429** | **0.621** | **0.399** | 0.162 | 0.075 | 0.064 | -0.034 | 0.191 | **0.704** | **0.432** | **0.528** | **0.412** | **0.578** | **0.483** | **0.511** | -0.256 | **0.462** | **1** | **0.959** | **0.870** | 0.126 | **0.753** | **0.784** | -0.208 | **0.350** | **0.571** | 0.220 | **0.310** | **0.474** | **0.439** |
| SM18:1/20:0 | **0.429** | 0.176 | **0.536** | **0.588** | **0.511** | 0.105 | 0.086 | -0.046 | -0.178 | **0.295** | **0.637** | **0.339** | **0.478** | **0.351** | **0.562** | **0.578** | **0.407** | **-0.303** | **0.341** | **0.959** | **1** | **0.947** | 0.226 | **0.779** | **0.877** | -0.208 | **0.402** | **0.655** | 0.224 | **0.311** | **0.387** | **0.520** |
| SM18:1/22:0 | **0.489** | 0.166 | **0.712** | **0.604** | **0.648** | 0.072 | 0.088 | -0.113 | -0.266 | **0.408** | **0.566** | 0.229 | **0.455** | **0.307** | **0.620** | **0.721** | **0.364** | **-0.387** | 0.240 | **0.870** | **0.947** | **1** | 0.265 | **0.824** | **0.967** | -0.193 | **0.426** | **0.750** | 0.251 | 0.237 | **0.292** | **0.633** |
| Glu18:1/24:1 | **0.316** | **0.381** | **0.339** | **0.407** | **0.432** | 0.008 | 0.112 | 0.119 | 0.167 | **0.684** | 0.224 | 0.016 | 0.124 | 0.268 | 0.280 | **0.354** | 0.081 | **0.438** | 0.199 | 0.126 | 0.226 | 0.265 | **1** | **0.401** | **0.307** | **0.445** | **0.497** | **0.472** | **0.331** | **0.439** | 0.139 | **0.381** |
| SM18:1/24:1 | **0.571** | 0.167 | **0.541** | **0.526** | **0.533** | 0.062 | 0.132 | 0.131 | -0.011 | **0.378** | **0.355** | 0.013 | 0.167 | 0.234 | **0.387** | **0.497** | **0.706** | -0.033 | **0.608** | **0.753** | **0.779** | **0.824** | **0.401** | **1** | **0.845** | 0.119 | 0.190 | **0.508** | 0.123 | 0.176 | 0.181 | **0.366** |
| SM18:1/24:0 | **0.497** | 0.138 | **0.744** | **0.558** | **0.629** | 0.065 | 0.141 | -0.085 | -0.279 | **0.430** | **0.483** | 0.061 | **0.302** | 0.166 | **0.556** | **0.734** | **0.370** | **-0.327** | 0.227 | **0.784** | **0.877** | **0.967** | **0.307** | **0.845** | **1** | -0.059 | **0.422** | **0.724** | 0.157 | 0.098 | 0.132 | **0.579** |
| Lct18:1/16:0 | 0.116 | 0.068 | -0.221 | -0.047 | -0.198 | -0.128 | -0.037 | **0.659** | **0.445** | **0.405** | -0.035 | **-0.316** | **-0.371** | -0.261 | -0.212 | -0.147 | 0.266 | **0.787** | **0.388** | -0.208 | -0.208 | -0.193 | **0.445** | 0.119 | -0.059 | **1** | -0.022 | -0.063 | -0.062 | -0.065 | -0.177 | -0.250 |
| 18:1/18:1 | **0.490** | **0.701** | **0.518** | **0.711** | **0.435** | 0.125 | 0.144 | -0.001 | 0.085 | **0.516** | **0.664** | **0.287** | **0.494** | **0.300** | **0.651** | **0.601** | -0.201 | -0.139 | -0.156 | **0.350** | **0.402** | **0.426** | **0.497** | 0.190 | **0.422** | -0.022 | **1** | **0.745** | **0.429** | 0.247 | 0.274 | **0.621** |
| 18:0/18:0 | **0.553** | **0.467** | **0.764** | **0.726** | **0.726** | -0.089 | -0.014 | -0.096 | -0.131 | **0.742** | **0.679** | **0.289** | **0.556** | **0.385** | **0.742** | **0.902** | 0.002 | **-0.302** | -0.056 | **0.571** | **0.655** | **0.750** | **0.472** | **0.508** | **0.724** | -0.063 | **0.745** | **1** | **0.589** | **0.334** | **0.334** | **0.869** |
| 18:0/20:0 | **0.403** | **0.517** | **0.378** | **0.424** | **0.478** | -0.116 | -0.045 | -0.011 | 0.044 | **0.480** | **0.389** | **0.695** | **0.760** | **0.767** | **0.704** | **0.580** | -0.047 | -0.178 | -0.070 | 0.220 | 0.224 | 0.251 | **0.331** | 0.123 | 0.157 | -0.062 | **0.429** | **0.589** | **1** | **0.612** | **0.711** | **0.775** |
| 18:1/22:2 | 0.233 | **0.391** | 0.182 | **0.387** | **0.349** | -0.040 | -0.155 | 0.138 | 0.156 | **0.431** | **0.317** | **0.690** | **0.617** | **0.760** | **0.414** | 0.190 | 0.109 | 0.125 | 0.197 | **0.310** | **0.311** | 0.237 | **0.439** | 0.176 | 0.098 | -0.065 | 0.247 | **0.334** | **0.612** | **1** | **0.776** | **0.427** |
| 18:1/22:1 | **0.376** | **0.384** | 0.155 | **0.453** | **0.293** | 0.154 | -0.014 | 0.238 | 0.247 | 0.199 | **0.514** | **0.934** | **0.850** | **0.901** | **0.628** | 0.236 | 0.258 | -0.079 | 0.261 | **0.474** | **0.387** | **0.292** | 0.139 | 0.181 | 0.132 | -0.177 | 0.274 | **0.334** | **0.711** | **0.776** | **1** | **0.481** |
| 18:0/22:0 | **0.475** | **0.385** | **0.781** | **0.580** | **0.793** | -0.069 | 0.032 | **-0.295** | **-0.311** | **0.565** | **0.465** | **0.464** | **0.705** | **0.603** | **0.804** | **0.920** | -0.078 | **-0.504** | -0.209 | **0.439** | **0.520** | **0.633** | **0.381** | **0.366** | **0.579** | -0.250 | **0.621** | **0.869** | **0.775** | **0.427** | **0.481** | **1** |
| 18:1/23:1 | **0.390** | **0.476** | 0.229 | **0.498** | **0.393** | 0.063 | -0.055 | 0.170 | 0.239 | **0.400** | **0.427** | **0.815** | **0.780** | **0.874** | **0.602** | **0.316** | 0.166 | -0.010 | 0.218 | **0.359** | **0.332** | 0.275 | **0.347** | 0.201 | 0.129 | -0.115 | **0.330** | **0.430** | **0.774** | **0.843** | **0.905** | **0.565** |
| 18:1/23:0 | **0.567** | **0.541** | **0.604** | **0.730** | **0.608** | 0.130 | 0.060 | -0.035 | 0.054 | **0.555** | **0.668** | **0.630** | **0.856** | **0.711** | **0.926** | **0.723** | -0.013 | **-0.287** | -0.008 | **0.493** | **0.529** | **0.594** | **0.440** | **0.347** | **0.511** | -0.200 | **0.714** | **0.809** | **0.754** | **0.539** | **0.635** | **0.861** |
| 18:0/23:0 | **0.433** | **0.286** | **0.882** | **0.554** | **0.862** | -0.116 | 0.039 | **-0.379** | **-0.446** | **0.614** | **0.384** | 0.180 | **0.463** | **0.347** | **0.658** | **0.961** | -0.078 | **-0.528** | -0.239 | **0.450** | **0.585** | **0.734** | **0.393** | **0.482** | **0.731** | -0.221 | **0.595** | **0.899** | **0.538** | 0.251 | 0.219 | **0.924** |
| 18:1/24:2 | **0.319** | **0.384** | 0.257 | **0.408** | **0.420** | 0.095 | -0.009 | 0.022 | 0.148 | **0.300** | **0.328** | **0.783** | **0.772** | **0.921** | **0.589** | **0.286** | 0.097 | -0.066 | 0.133 | 0.284 | 0.258 | 0.223 | **0.393** | 0.156 | 0.080 | -0.228 | 0.280 | **0.352** | **0.723** | **0.888** | **0.827** | **0.569** |
| 18:1/25:1 | **0.687** | **0.488** | **0.667** | **0.704** | **0.657** | 0.121 | 0.127 | 0.096 | 0.073 | **0.599** | **0.611** | **0.505** | **0.728** | **0.703** | **0.899** | **0.788** | 0.215 | -0.236 | 0.169 | **0.548** | **0.576** | **0.666** | **0.457** | **0.531** | **0.636** | -0.062 | **0.615** | **0.802** | **0.675** | **0.494** | **0.595** | **0.843** |
| 18:1/26:2 | **0.557** | **0.527** | **0.385** | **0.575** | **0.470** | 0.005 | 0.007 | 0.122 | **0.331** | **0.514** | **0.523** | **0.510** | **0.611** | **0.721** | **0.663** | **0.509** | 0.151 | 0.023 | 0.209 | **0.327** | 0.270 | **0.289** | **0.518** | **0.304** | 0.230 | 0.004 | **0.476** | **0.585** | **0.678** | **0.557** | **0.582** | **0.622** |
| 18:1/26:1 | **0.692** | **0.557** | **0.487** | **0.654** | **0.463** | 0.076 | 0.142 | 0.233 | **0.328** | **0.533** | **0.609** | **0.290** | **0.469** | **0.545** | **0.724** | **0.654** | 0.265 | -0.015 | 0.266 | **0.420** | **0.367** | **0.434** | **0.451** | **0.477** | **0.449** | 0.117 | **0.597** | **0.675** | **0.524** | 0.237 | **0.405** | **0.608** |
| 18:1/26:0 | **0.382** | **0.506** | 0.226 | **0.429** | 0.144 | -0.066 | -0.051 | **0.382** | **0.322** | **0.471** | **0.399** | **0.401** | **0.427** | **0.444** | **0.551** | **0.394** | 0.187 | 0.162 | 0.202 | 0.235 | 0.184 | 0.217 | 0.277 | 0.257 | 0.236 | **0.332** | **0.354** | **0.430** | **0.623** | **0.347** | **0.437** | **0.391** |
| SM18:0/16:0 | **0.537** | 0.200 | **0.683** | **0.584** | **0.720** | 0.112 | 0.161 | -0.109 | -0.215 | **0.352** | **0.354** | **0.325** | **0.453** | **0.499** | **0.529** | **0.600** | **0.526** | **-0.305** | **0.402** | **0.787** | **0.823** | **0.832** | **0.333** | **0.809** | **0.774** | -0.196 | 0.270 | **0.597** | **0.347** | **0.469** | **0.443** | **0.605** |
| Hex18:1/18:0 | 0.249 | **0.437** | 0.151 | **0.607** | 0.223 | 0.027 | -0.060 | 0.156 | 0.267 | **0.530** | **0.634** | **0.320** | **0.385** | **0.327** | **0.407** | 0.262 | 0.150 | **0.320** | **0.351** | **0.532** | **0.530** | **0.426** | **0.611** | **0.392** | **0.333** | 0.173 | **0.463** | **0.533** | **0.352** | **0.514** | **0.358** | **0.297** |
| SM18:0/18:0 | -0.118 | -0.088 | 0.084 | -0.132 | 0.065 | -0.164 | -0.124 | -0.102 | -0.200 | **0.294** | 0.136 | -0.027 | -0.020 | -0.030 | 0.018 | 0.131 | -0.034 | 0.077 | 0.056 | 0.216 | **0.305** | 0.283 | 0.224 | 0.214 | 0.271 | 0.054 | -0.022 | 0.177 | 0.051 | 0.191 | -0.022 | 0.049 |
| SM18:0/20:0 | **0.333** | 0.200 | **0.520** | **0.441** | **0.523** | -0.049 | 0.014 | -0.120 | -0.230 | **0.404** | **0.373** | 0.123 | 0.272 | 0.211 | **0.394** | **0.576** | **0.333** | -0.258 | 0.242 | **0.687** | **0.756** | **0.777** | 0.182 | **0.683** | **0.718** | -0.123 | 0.198 | **0.615** | **0.300** | 0.204 | 0.216 | **0.478** |
| SM18:1/22:2 | **0.377** | 0.152 | **0.374** | **0.459** | **0.391** | -0.016 | 0.056 | 0.077 | -0.045 | 0.256 | 0.262 | 0.144 | 0.211 | 0.276 | **0.302** | **0.323** | **0.696** | -0.006 | **0.610** | **0.737** | **0.731** | **0.708** | 0.284 | **0.874** | **0.667** | 0.042 | 0.037 | **0.366** | 0.178 | **0.330** | 0.265 | 0.263 |
| Hex18:1/22:0 | **0.378** | **0.449** | **0.416** | **0.656** | **0.485** | 0.070 | 0.083 | 0.051 | 0.141 | **0.644** | **0.528** | 0.251 | **0.398** | **0.363** | **0.503** | **0.457** | 0.052 | 0.225 | 0.153 | **0.368** | **0.445** | **0.447** | **0.865** | **0.415** | **0.429** | 0.215 | **0.678** | **0.657** | **0.418** | **0.484** | **0.295** | **0.514** |
| SM18:1/22:1 | **0.559** | 0.152 | **0.476** | **0.495** | **0.496** | 0.097 | 0.138 | 0.131 | -0.003 | **0.290** | **0.364** | 0.077 | 0.205 | 0.258 | **0.372** | **0.431** | **0.734** | -0.060 | **0.632** | **0.796** | **0.809** | **0.824** | **0.319** | **0.979** | **0.820** | 0.038 | 0.147 | **0.446** | 0.106 | 0.202 | 0.242 | **0.324** |
| SM18:0/22:0 | **0.437** | 0.183 | **0.832** | **0.544** | **0.799** | -0.031 | 0.088 | **-0.310** | **-0.465** | **0.449** | **0.425** | 0.206 | **0.444** | **0.325** | **0.603** | **0.853** | 0.173 | **-0.538** | -0.015 | **0.716** | **0.836** | **0.927** | 0.268 | **0.688** | **0.904** | **-0.286** | **0.459** | **0.816** | **0.370** | 0.254 | 0.260 | **0.786** |
| SM18:1/23:1 | **0.485** | 0.216 | **0.494** | **0.502** | **0.537** | -0.072 | 0.011 | 0.028 | -0.056 | **0.347** | **0.292** | 0.127 | 0.228 | **0.306** | **0.332** | **0.454** | **0.611** | -0.107 | **0.510** | **0.703** | **0.710** | **0.740** | 0.247 | **0.866** | **0.699** | -0.024 | 0.074 | **0.478** | 0.246 | 0.271 | 0.282 | **0.375** |
| SM18:1/23:0 | **0.502** | 0.170 | **0.791** | **0.592** | **0.712** | 0.039 | 0.097 | -0.172 | **-0.315** | **0.452** | **0.458** | 0.129 | **0.373** | 0.262 | **0.582** | **0.765** | **0.350** | **-0.398** | 0.199 | **0.790** | **0.886** | **0.979** | **0.311** | **0.844** | **0.975** | -0.172 | **0.410** | **0.762** | 0.246 | 0.202 | 0.207 | **0.656** |
| SM18:0/23:0 | **0.397** | 0.162 | **0.870** | **0.511** | **0.843** | -0.061 | 0.067 | **-0.368** | **-0.513** | **0.507** | **0.364** | 0.134 | **0.385** | 0.260 | **0.551** | **0.867** | 0.059 | **-0.534** | -0.126 | **0.602** | **0.750** | **0.871** | **0.329** | **0.617** | **0.868** | -0.257 | **0.496** | **0.824** | **0.341** | 0.231 | 0.183 | **0.798** |
| SM18:1/24:3 | 0.110 | **0.502** | 0.099 | 0.217 | 0.103 | 0.168 | 0.146 | 0.092 | 0.165 | 0.131 | -0.005 | 0.248 | 0.230 | 0.216 | 0.167 | -0.032 | 0.079 | 0.108 | 0.043 | -0.036 | -0.035 | -0.003 | 0.164 | 0.055 | -0.018 | 0.019 | 0.140 | 0.070 | **0.302** | 0.275 | 0.250 | 0.070 |
| SM18:1/24:2 | **0.517** | 0.165 | **0.529** | **0.518** | **0.541** | 0.046 | 0.103 | 0.077 | -0.030 | **0.396** | **0.292** | 0.033 | 0.170 | 0.259 | **0.342** | **0.448** | **0.693** | 0.015 | **0.613** | **0.713** | **0.748** | **0.781** | **0.476** | **0.971** | **0.785** | 0.114 | 0.157 | **0.480** | 0.146 | **0.289** | 0.195 | **0.366** |
| Hex18:1/24:0 | **0.324** | 0.211 | **0.569** | **0.464** | **0.526** | -0.041 | 0.070 | 0.007 | -0.130 | **0.700** | **0.368** | -0.114 | 0.088 | -0.021 | **0.334** | **0.588** | 0.024 | 0.126 | 0.023 | **0.308** | **0.461** | **0.551** | **0.768** | **0.490** | **0.640** | **0.397** | **0.606** | **0.674** | 0.190 | 0.123 | -0.047 | **0.481** |
| SM18:0/24:1 | **0.558** | 0.215 | **0.778** | **0.540** | **0.784** | 0.019 | 0.172 | -0.138 | -0.265 | **0.412** | **0.318** | 0.077 | 0.268 | **0.308** | **0.476** | **0.715** | **0.422** | **-0.320** | 0.246 | **0.690** | **0.773** | **0.861** | **0.383** | **0.870** | **0.888** | -0.106 | **0.349** | **0.664** | 0.258 | 0.225 | 0.205 | **0.610** |
| SM18:0/24:0 | **0.434** | 0.157 | **0.810** | **0.527** | **0.739** | -0.049 | 0.100 | -0.228 | **-0.427** | **0.478** | **0.428** | 0.073 | **0.310** | 0.179 | **0.532** | **0.834** | 0.197 | **-0.437** | 0.017 | **0.695** | **0.824** | **0.924** | **0.305** | **0.727** | **0.950** | -0.143 | **0.472** | **0.795** | 0.251 | 0.133 | 0.135 | **0.694** |
| SM18:1/25:2 | **0.547** | 0.140 | **0.603** | **0.490** | **0.586** | 0.067 | 0.137 | 0.012 | -0.107 | **0.322** | **0.295** | 0.075 | 0.249 | 0.284 | **0.429** | **0.516** | **0.648** | -0.172 | **0.507** | **0.735** | **0.778** | **0.849** | **0.334** | **0.953** | **0.853** | -0.030 | 0.162 | **0.496** | 0.151 | 0.234 | 0.212 | **0.430** |
| SM18:1/25:1 | **0.305** | 0.090 | **0.475** | **0.291** | **0.447** | -0.007 | 0.071 | -0.002 | -0.134 | **0.307** | 0.174 | -0.079 | 0.036 | 0.096 | 0.222 | **0.418** | **0.512** | -0.128 | **0.384** | **0.536** | **0.590** | **0.673** | 0.156 | **0.772** | **0.695** | 0.026 | -0.033 | **0.375** | 0.061 | 0.054 | 0.051 | 0.242 |
| SM18:1/25:0 | **0.481** | 0.121 | **0.722** | **0.514** | **0.609** | 0.031 | 0.123 | -0.038 | -0.259 | **0.452** | **0.405** | -0.039 | 0.183 | 0.070 | **0.448** | **0.681** | **0.414** | -0.248 | 0.257 | **0.722** | **0.821** | **0.924** | **0.308** | **0.855** | **0.983** | 0.023 | **0.355** | **0.675** | 0.081 | 0.062 | 0.054 | **0.497** |
| SM18:1/26:3 | 0.184 | 0.115 | 0.026 | 0.066 | 0.072 | 0.069 | 0.053 | 0.141 | 0.102 | -0.110 | 0.000 | **0.335** | **0.313** | **0.321** | 0.218 | -0.037 | 0.188 | -0.084 | 0.073 | 0.145 | 0.128 | 0.147 | -0.035 | 0.166 | 0.128 | -0.144 | -0.030 | -0.028 | 0.239 | 0.235 | **0.351** | 0.090 |
| SM18:1/26:2 | **0.560** | 0.167 | **0.562** | **0.523** | **0.550** | 0.067 | 0.117 | 0.083 | -0.028 | **0.314** | **0.393** | 0.120 | 0.271 | **0.322** | **0.450** | **0.501** | **0.662** | -0.132 | **0.550** | **0.785** | **0.804** | **0.840** | **0.331** | **0.941** | **0.835** | -0.010 | 0.194 | **0.506** | 0.159 | 0.243 | 0.259 | **0.410** |
| SM18:1/26:1 | **0.579** | 0.151 | **0.548** | **0.496** | **0.517** | 0.064 | 0.149 | 0.148 | -0.025 | **0.345** | **0.423** | 0.006 | 0.173 | 0.194 | **0.416** | **0.527** | **0.636** | -0.093 | **0.526** | **0.780** | **0.812** | **0.859** | **0.331** | **0.957** | **0.897** | 0.083 | 0.251 | **0.528** | 0.066 | 0.101 | 0.153 | **0.366** |
| SM18:1/26:0 | **0.461** | 0.086 | **0.555** | **0.474** | **0.453** | 0.051 | 0.109 | 0.102 | -0.139 | **0.369** | **0.481** | -0.029 | 0.166 | 0.034 | **0.413** | **0.555** | **0.472** | -0.141 | **0.361** | **0.777** | **0.849** | **0.907** | 0.274 | **0.856** | **0.955** | 0.099 | **0.322** | **0.588** | -0.005 | 0.018 | 0.057 | **0.367** |
| Lac18:1/18:0 | **0.418** | 0.275 | **0.300** | **0.532** | **0.311** | 0.278 | 0.247 | 0.083 | 0.181 | 0.080 | **0.475** | **0.480** | **0.571** | **0.518** | **0.605** | **0.372** | 0.106 | -0.132 | 0.079 | **0.348** | **0.287** | 0.274 | **0.306** | 0.204 | 0.251 | -0.017 | **0.537** | **0.392** | **0.398** | 0.205 | **0.468** | **0.483** |

*Values in bold are significant ($P<0.05$)

**Table S3.** *(continued)* Spearman correlation measured among different sphingolipids *.

| Variables | 18:1/23:1 | 18:1/23:0 | 18:0/23:0 | 18:1/24:2 | 18:1/25:1 | 18:1/26:2 | 18:1/26:1 | 18:1/26:0 | SM18:0/16:0 | Hex18:1/18:0 | SM18:0/18:0 | SM18:0/20:0 | SM18:1/22:2 | Hex18:1/22:0 | SM18:1/22:1 | SM18:0/22:0 | SM18:1/23:1 | SM18:1/23:0 | SM18:0/23:0 | SM18:1/24:3 | SM18:1/24:2 | Hex18:1/24:0 | SM18:0/24:1 | SM18:0/24:0 | SM18:1/25:2 | SM18:1/25:1 | SM18:1/25:0 | SM18:1/26:3 | SM18:1/26:2 | SM18:1/26:1 | SM18:1/26:0 | Lac18:1/18:0 |
|---|---|---|---|---|---|---|---|---|---|---|---|---|---|---|---|---|---|---|---|---|---|---|---|---|---|---|---|---|---|---|---|---|
| dSa | **0.390** | **0.567** | **0.433** | **0.319** | **0.687** | **0.557** | **0.692** | **0.382** | **0.537** | 0.249 | -0.118 | **0.333** | **0.377** | **0.378** | **0.559** | **0.437** | **0.485** | **0.502** | **0.397** | 0.110 | **0.517** | **0.324** | **0.558** | **0.434** | **0.547** | **0.305** | **0.481** | 0.184 | **0.560** | **0.579** | **0.461** | **0.418** |
| So | **0.476** | **0.541** | **0.286** | **0.384** | **0.488** | **0.527** | **0.557** | **0.506** | 0.200 | **0.437** | -0.088 | 0.200 | 0.152 | **0.449** | 0.152 | 0.183 | 0.216 | 0.170 | 0.162 | **0.502** | 0.165 | 0.211 | 0.215 | 0.157 | 0.140 | 0.090 | 0.121 | 0.115 | 0.167 | 0.151 | 0.086 | 0.275 |
| Sa | 0.229 | **0.604** | **0.882** | 0.257 | **0.667** | **0.385** | **0.487** | 0.226 | **0.683** | 0.151 | 0.084 | **0.520** | **0.374** | **0.416** | **0.476** | **0.832** | **0.494** | **0.791** | **0.870** | 0.099 | **0.529** | **0.569** | **0.778** | **0.810** | **0.603** | **0.475** | **0.722** | 0.026 | **0.562** | **0.548** | **0.555** | **0.300** |
| 18:1/2:0 | **0.498** | **0.730** | **0.554** | **0.408** | **0.704** | **0.575** | **0.654** | **0.429** | **0.584** | **0.607** | -0.132 | **0.441** | **0.459** | **0.656** | **0.495** | **0.544** | **0.502** | **0.592** | **0.511** | 0.217 | **0.518** | **0.464** | **0.540** | **0.527** | **0.490** | **0.291** | **0.514** | 0.066 | **0.523** | **0.496** | **0.474** | **0.532** |
| 18:0/2:0 | **0.393** | **0.608** | **0.862** | **0.420** | **0.657** | **0.470** | **0.463** | 0.144 | **0.720** | 0.223 | 0.065 | **0.523** | **0.391** | **0.485** | **0.496** | **0.799** | **0.537** | **0.712** | **0.843** | 0.103 | **0.541** | **0.526** | **0.784** | **0.739** | **0.586** | **0.447** | **0.609** | 0.072 | **0.550** | **0.517** | **0.453** | **0.311** |
| GluSo | 0.063 | 0.130 | -0.116 | 0.095 | 0.121 | 0.005 | 0.076 | -0.066 | 0.112 | 0.027 | -0.164 | -0.049 | -0.016 | 0.070 | 0.097 | -0.031 | -0.072 | 0.039 | -0.061 | 0.168 | 0.046 | -0.041 | 0.019 | -0.049 | 0.067 | -0.007 | 0.031 | 0.069 | 0.067 | 0.064 | 0.051 | 0.278 |
| LysoSM | -0.055 | 0.060 | 0.039 | -0.009 | 0.127 | 0.007 | 0.142 | -0.051 | 0.161 | -0.060 | -0.124 | 0.014 | 0.056 | 0.083 | 0.138 | 0.088 | 0.011 | 0.097 | 0.067 | 0.146 | 0.103 | 0.070 | 0.172 | 0.100 | 0.137 | 0.071 | 0.123 | 0.053 | 0.117 | 0.149 | 0.109 | 0.247 |
| 18:1/14:0 | 0.170 | -0.035 | **-0.379** | 0.022 | 0.096 | 0.122 | 0.233 | **0.382** | -0.109 | 0.156 | -0.102 | -0.120 | 0.077 | 0.051 | 0.131 | **-0.310** | 0.028 | -0.172 | **-0.368** | 0.092 | 0.077 | 0.007 | -0.138 | -0.228 | 0.012 | -0.002 | -0.038 | 0.141 | 0.083 | 0.148 | 0.102 | 0.083 |
| 18:1/16:0 | 0.239 | 0.054 | **-0.446** | 0.148 | 0.073 | **0.331** | **0.328** | **0.322** | -0.215 | 0.267 | -0.200 | -0.230 | -0.045 | 0.141 | -0.003 | **-0.465** | -0.056 | **-0.315** | **-0.513** | 0.165 | -0.030 | -0.130 | -0.265 | **-0.427** | -0.107 | -0.134 | -0.259 | 0.102 | -0.028 | -0.025 | -0.139 | 0.181 |
| 18:0/16:0 | **0.400** | **0.555** | **0.614** | **0.300** | **0.599** | **0.514** | **0.533** | **0.471** | **0.352** | **0.530** | **0.294** | **0.404** | 0.256 | **0.644** | **0.290** | **0.449** | **0.347** | **0.452** | **0.507** | 0.131 | **0.396** | **0.700** | **0.412** | **0.478** | **0.322** | **0.307** | **0.452** | -0.110 | **0.314** | **0.345** | **0.369** | 0.080 |
| 18:1/18:0 | **0.427** | **0.668** | **0.384** | **0.328** | **0.611** | **0.523** | **0.609** | **0.399** | **0.354** | **0.634** | 0.136 | **0.373** | 0.262 | **0.528** | **0.364** | **0.425** | **0.292** | **0.458** | **0.364** | -0.005 | **0.292** | **0.368** | **0.318** | **0.428** | **0.295** | 0.174 | **0.405** | 0.000 | **0.393** | **0.423** | **0.481** | **0.475** |
| 18:1/20:0 | **0.815** | **0.630** | 0.180 | **0.783** | **0.505** | **0.510** | **0.290** | **0.401** | **0.325** | **0.320** | -0.027 | 0.123 | 0.144 | 0.251 | 0.077 | 0.206 | 0.127 | 0.129 | 0.134 | 0.248 | 0.033 | -0.114 | 0.077 | 0.073 | 0.075 | -0.079 | -0.039 | **0.335** | 0.120 | 0.006 | -0.029 | **0.480** |
| 18:1/22:0 | **0.780** | **0.856** | **0.463** | **0.772** | **0.728** | **0.611** | **0.469** | **0.427** | **0.453** | **0.385** | -0.020 | 0.272 | 0.211 | **0.398** | 0.205 | **0.444** | 0.228 | **0.373** | **0.385** | 0.230 | 0.170 | 0.088 | 0.268 | **0.310** | 0.249 | 0.036 | 0.183 | **0.313** | 0.271 | 0.173 | 0.166 | **0.571** |
| 18:1/24:1 | **0.874** | **0.711** | **0.347** | **0.921** | **0.703** | **0.721** | **0.545** | **0.444** | **0.499** | **0.327** | -0.030 | 0.211 | 0.276 | **0.363** | 0.258 | **0.325** | **0.306** | 0.262 | 0.260 | 0.216 | 0.259 | -0.021 | **0.308** | 0.179 | 0.284 | 0.096 | 0.070 | **0.321** | **0.322** | 0.194 | 0.034 | **0.518** |
| 18:1/24:0 | **0.602** | **0.926** | **0.658** | **0.589** | **0.899** | **0.663** | **0.724** | **0.551** | **0.529** | **0.407** | 0.018 | **0.394** | **0.302** | **0.503** | **0.372** | **0.603** | **0.332** | **0.582** | **0.551** | 0.167 | **0.342** | **0.334** | **0.476** | **0.532** | **0.429** | 0.222 | **0.448** | 0.218 | **0.450** | **0.416** | **0.413** | **0.605** |
| 18:0/24:0 | **0.316** | **0.723** | **0.961** | **0.286** | **0.788** | **0.509** | **0.654** | **0.394** | **0.600** | 0.262 | 0.131 | **0.576** | **0.323** | **0.457** | **0.431** | **0.853** | **0.454** | **0.765** | **0.867** | -0.032 | **0.448** | **0.588** | **0.715** | **0.834** | **0.516** | **0.418** | **0.681** | -0.037 | **0.501** | **0.527** | **0.555** | **0.372** |
| SM18:1/14:0 | 0.166 | -0.013 | -0.078 | 0.097 | 0.215 | 0.151 | 0.265 | 0.187 | **0.526** | 0.150 | -0.034 | **0.333** | **0.696** | 0.052 | **0.734** | 0.173 | **0.611** | **0.350** | 0.059 | 0.079 | **0.693** | 0.024 | **0.422** | 0.197 | **0.648** | **0.512** | **0.414** | 0.188 | **0.662** | **0.636** | **0.472** | 0.106 |
| Glc18:1/16:0 | -0.010 | **-0.287** | **-0.528** | -0.066 | -0.236 | 0.023 | -0.015 | 0.162 | **-0.305** | **0.320** | 0.077 | -0.258 | -0.006 | 0.225 | -0.060 | **-0.538** | -0.107 | **-0.398** | **-0.534** | 0.108 | 0.015 | 0.126 | **-0.320** | **-0.437** | -0.172 | -0.128 | -0.248 | -0.084 | -0.132 | -0.093 | -0.141 | -0.132 |
| SM18:1/16:0 | 0.218 | -0.008 | -0.239 | 0.133 | 0.169 | 0.209 | 0.266 | 0.202 | **0.402** | **0.351** | 0.056 | 0.242 | **0.610** | 0.153 | **0.632** | -0.015 | **0.510** | 0.199 | -0.126 | 0.043 | **0.613** | 0.023 | 0.246 | 0.017 | **0.507** | **0.384** | 0.257 | 0.073 | **0.550** | **0.526** | **0.361** | 0.079 |
| SM18:1/18:0 | **0.359** | **0.493** | **0.450** | 0.284 | **0.548** | **0.327** | **0.420** | 0.235 | **0.787** | **0.532** | 0.216 | **0.687** | **0.737** | **0.368** | **0.796** | **0.716** | **0.703** | **0.790** | **0.602** | -0.036 | **0.713** | **0.308** | **0.690** | **0.695** | **0.735** | **0.536** | **0.722** | 0.145 | **0.785** | **0.780** | **0.777** | **0.348** |
| SM18:1/20:0 | **0.332** | **0.529** | **0.585** | 0.258 | **0.576** | 0.270 | **0.367** | 0.184 | **0.823** | **0.530** | **0.305** | **0.756** | **0.731** | **0.445** | **0.809** | **0.836** | **0.710** | **0.886** | **0.750** | -0.035 | **0.748** | **0.461** | **0.773** | **0.824** | **0.778** | **0.590** | **0.821** | 0.128 | **0.804** | **0.812** | **0.849** | **0.287** |
| SM18:1/22:0 | 0.275 | **0.594** | **0.734** | 0.223 | **0.666** | **0.289** | **0.434** | 0.217 | **0.832** | **0.426** | 0.283 | **0.777** | **0.708** | **0.447** | **0.824** | **0.927** | **0.740** | **0.979** | **0.871** | -0.003 | **0.781** | **0.551** | **0.861** | **0.924** | **0.849** | **0.673** | **0.924** | 0.147 | **0.840** | **0.859** | **0.907** | 0.274 |
| Glu18:1/24:1 | **0.347** | **0.440** | **0.393** | **0.393** | **0.457** | **0.518** | **0.451** | 0.277 | **0.333** | **0.611** | 0.224 | 0.182 | 0.284 | **0.865** | **0.319** | 0.268 | 0.247 | **0.311** | **0.329** | 0.164 | **0.476** | **0.768** | **0.383** | **0.305** | **0.334** | 0.156 | **0.308** | -0.035 | **0.331** | **0.331** | 0.274 | **0.306** |
| SM18:1/24:1 | 0.201 | **0.347** | **0.482** | 0.156 | **0.531** | **0.304** | **0.477** | 0.257 | **0.809** | **0.392** | 0.214 | **0.683** | **0.874** | **0.415** | **0.979** | **0.688** | **0.866** | **0.844** | **0.617** | 0.055 | **0.971** | **0.490** | **0.870** | **0.727** | **0.953** | **0.772** | **0.855** | 0.166 | **0.941** | **0.957** | **0.856** | 0.204 |
| SM18:1/24:0 | 0.129 | **0.511** | **0.731** | 0.080 | **0.636** | 0.230 | **0.449** | 0.236 | **0.774** | **0.333** | 0.271 | **0.718** | **0.667** | **0.429** | **0.820** | **0.904** | **0.699** | **0.975** | **0.868** | -0.018 | **0.785** | **0.640** | **0.888** | **0.950** | **0.853** | **0.695** | **0.983** | 0.128 | **0.835** | **0.897** | **0.955** | 0.251 |
| Lct18:1/16:0 | -0.115 | -0.200 | -0.221 | -0.228 | -0.062 | 0.004 | 0.117 | **0.332** | -0.196 | 0.173 | 0.054 | -0.123 | 0.042 | 0.215 | 0.038 | **-0.286** | -0.024 | -0.172 | -0.257 | 0.019 | 0.114 | **0.397** | -0.106 | -0.143 | -0.030 | 0.026 | 0.023 | -0.144 | -0.010 | 0.083 | 0.099 | -0.017 |
| 18:1/18:1 | **0.330** | **0.714** | **0.595** | 0.280 | **0.615** | **0.476** | **0.597** | **0.354** | 0.270 | **0.463** | -0.022 | 0.198 | 0.037 | **0.678** | 0.147 | **0.459** | 0.074 | **0.410** | **0.496** | 0.140 | 0.157 | **0.606** | **0.349** | **0.472** | 0.162 | -0.033 | **0.355** | -0.030 | 0.194 | 0.251 | **0.322** | **0.537** |
| 18:0/18:0 | **0.430** | **0.809** | **0.899** | **0.352** | **0.802** | **0.585** | **0.675** | **0.430** | **0.597** | **0.533** | 0.177 | **0.615** | **0.366** | **0.657** | **0.446** | **0.816** | **0.478** | **0.762** | **0.824** | 0.070 | **0.480** | **0.674** | **0.664** | **0.795** | **0.496** | **0.375** | **0.675** | -0.028 | **0.506** | **0.528** | **0.588** | **0.392** |
| 18:0/20:0 | **0.774** | **0.754** | **0.538** | **0.723** | **0.675** | **0.678** | **0.524** | **0.623** | **0.347** | **0.352** | 0.051 | **0.300** | 0.178 | **0.418** | 0.106 | **0.370** | 0.246 | 0.246 | **0.341** | **0.302** | 0.146 | 0.190 | 0.258 | 0.251 | 0.151 | 0.061 | 0.081 | 0.239 | 0.159 | 0.066 | -0.005 | **0.398** |
| 18:1/22:2 | **0.843** | **0.539** | 0.251 | **0.888** | **0.494** | **0.557** | 0.237 | **0.347** | **0.469** | **0.514** | 0.191 | 0.204 | **0.330** | **0.484** | 0.202 | 0.254 | 0.271 | 0.202 | 0.231 | 0.275 | **0.289** | 0.123 | 0.225 | 0.133 | 0.234 | 0.054 | 0.062 | 0.235 | 0.243 | 0.101 | 0.018 | 0.205 |
| 18:1/22:1 | **0.905** | **0.635** | 0.219 | **0.827** | **0.595** | **0.582** | **0.405** | **0.437** | **0.443** | **0.358** | -0.022 | 0.216 | 0.265 | **0.295** | 0.242 | 0.260 | 0.282 | 0.207 | 0.183 | 0.250 | 0.195 | -0.047 | 0.205 | 0.135 | 0.212 | 0.051 | 0.054 | **0.351** | 0.259 | 0.153 | 0.057 | **0.468** |
| 18:0/22:0 | **0.565** | **0.861** | **0.924** | **0.569** | **0.843** | **0.622** | **0.608** | **0.391** | **0.605** | **0.297** | 0.049 | **0.478** | 0.263 | **0.514** | **0.324** | **0.786** | **0.375** | **0.656** | **0.798** | 0.070 | **0.366** | **0.481** | **0.610** | **0.694** | **0.430** | 0.242 | **0.497** | 0.090 | **0.410** | **0.366** | **0.367** | **0.483** |
| 18:1/23:1 | **1** | **0.727** | **0.337** | **0.879** | **0.675** | **0.651** | **0.421** | **0.460** | **0.455** | **0.442** | -0.021 | 0.261 | 0.270 | **0.452** | 0.220 | **0.286** | **0.341** | 0.243 | 0.244 | **0.323** | 0.256 | 0.096 | 0.236 | 0.158 | 0.227 | 0.073 | 0.064 | **0.301** | 0.232 | 0.115 | 0.013 | **0.400** |
| 18:1/23:0 | **0.727** | **1** | **0.725** | **0.676** | **0.914** | **0.690** | **0.654** | **0.469** | **0.526** | **0.483** | 0.021 | **0.395** | 0.255 | **0.644** | **0.322** | **0.615** | **0.331** | **0.576** | **0.605** | 0.243 | **0.344** | **0.445** | **0.467** | **0.530** | **0.393** | 0.162 | **0.412** | 0.215 | **0.386** | **0.339** | **0.346** | **0.568** |
| 18:0/23:0 | **0.337** | **0.725** | **1** | **0.322** | **0.755** | **0.457** | **0.544** | 0.265 | **0.646** | 0.262 | 0.117 | **0.572** | **0.322** | **0.505** | **0.420** | **0.893** | **0.460** | **0.788** | **0.931** | -0.012 | **0.461** | **0.617** | **0.745** | **0.855** | **0.526** | **0.403** | **0.686** | -0.013 | **0.490** | **0.499** | **0.536** | **0.330** |
| 18:1/24:2 | **0.879** | **0.676** | **0.322** | **1** | **0.634** | **0.719** | **0.398** | **0.330** | **0.460** | **0.366** | -0.001 | 0.112 | 0.234 | **0.461** | 0.177 | 0.264 | 0.231 | 0.197 | 0.238 | 0.259 | 0.253 | 0.038 | 0.247 | 0.110 | 0.253 | 0.001 | 0.006 | **0.307** | 0.258 | 0.088 | -0.049 | **0.447** |
| 18:1/25:1 | **0.675** | **0.914** | **0.755** | **0.634** | **1** | **0.731** | **0.785** | **0.525** | **0.637** | **0.369** | 0.019 | **0.437** | **0.359** | **0.572** | **0.489** | **0.681** | **0.460** | **0.673** | **0.659** | 0.150 | **0.503** | **0.476** | **0.630** | **0.631** | **0.562** | **0.338** | **0.566** | 0.209 | **0.562** | **0.542** | **0.496** | **0.557** |
| 18:1/26:2 | **0.651** | **0.690** | **0.457** | **0.719** | **0.731** | **1** | **0.832** | **0.525** | **0.410** | **0.472** | -0.019 | 0.231 | 0.234 | **0.564** | 0.272 | **0.311** | **0.332** | **0.298** | **0.294** | 0.204 | **0.341** | 0.250 | **0.365** | 0.230 | **0.327** | 0.175 | 0.179 | 0.120 | **0.394** | **0.292** | 0.127 | **0.556** |
| 18:1/26:1 | **0.421** | **0.654** | **0.544** | **0.398** | **0.785** | **0.832** | **1** | **0.602** | **0.409** | **0.370** | -0.070 | **0.317** | 0.261 | **0.488** | **0.417** | **0.423** | **0.403** | **0.452** | **0.394** | 0.066 | **0.407** | **0.364** | **0.512** | **0.421** | **0.418** | **0.324** | **0.407** | 0.014 | **0.502** | **0.515** | **0.373** | **0.570** |
| 18:1/26:0 | **0.460** | **0.469** | 0.265 | **0.330** | **0.525** | **0.525** | **0.602** | **1** | 0.184 | **0.352** | 0.033 | **0.306** | 0.259 | **0.303** | 0.194 | 0.183 | **0.296** | 0.227 | 0.123 | **0.359** | 0.219 | 0.229 | 0.217 | 0.193 | 0.192 | 0.264 | 0.230 | 0.240 | 0.242 | 0.247 | 0.207 | 0.259 |
| SM18:0/16:0 | **0.455** | **0.526** | **0.646** | **0.460** | **0.637** | **0.410** | **0.409** | 0.184 | **1** | **0.394** | 0.126 | **0.664** | **0.783** | **0.441** | **0.817** | **0.824** | **0.766** | **0.841** | **0.763** | 0.010 | **0.836** | **0.381** | **0.866** | **0.752** | **0.846** | **0.578** | **0.740** | 0.133 | **0.840** | **0.765** | **0.668** | **0.323** |
| Hex18:1/18:0 | **0.442** | **0.483** | 0.262 | **0.366** | **0.369** | **0.472** | **0.370** | **0.352** | **0.394** | **1** | **0.385** | **0.515** | **0.495** | **0.758** | **0.374** | **0.304** | **0.417** | **0.364** | 0.253 | 0.216 | **0.456** | **0.481** | 0.246 | 0.284 | **0.300** | 0.270 | **0.292** | -0.073 | **0.372** | **0.317** | **0.337** | 0.175 |
| SM18:0/18:0 | -0.021 | 0.021 | 0.117 | -0.001 | 0.019 | -0.019 | -0.070 | 0.033 | 0.126 | **0.385** | **1** | **0.443** | 0.254 | 0.162 | 0.217 | 0.222 | 0.188 | 0.241 | 0.205 | 0.087 | 0.226 | **0.302** | 0.114 | 0.251 | 0.178 | **0.386** | 0.265 | -0.105 | 0.192 | 0.224 | **0.297** | **-0.476** |
| SM18:0/20:0 | 0.261 | **0.395** | **0.572** | 0.112 | **0.437** | 0.231 | **0.317** | **0.306** | **0.664** | **0.515** | **0.443** | **1** | **0.687** | **0.287** | **0.669** | **0.753** | **0.795** | **0.772** | **0.662** | 0.210 | **0.662** | **0.377** | **0.643** | **0.717** | **0.643** | **0.817** | **0.701** | 0.056 | **0.635** | **0.640** | **0.657** | -0.125 |
| SM18:1/22:2 | 0.270 | 0.255 | **0.322** | 0.234 | **0.359** | 0.234 | 0.261 | 0.259 | **0.783** | **0.495** | 0.254 | **0.687** | **1** | **0.329** | **0.884** | **0.566** | **0.877** | **0.707** | **0.459** | 0.216 | **0.906** | 0.269 | **0.700** | **0.550** | **0.861** | **0.694** | **0.674** | 0.229 | **0.849** | **0.781** | **0.684** | 0.048 |
| Hex18:1/22:0 | **0.452** | **0.644** | **0.505** | **0.461** | **0.572** | **0.564** | **0.488** | **0.303** | **0.441** | **0.758** | 0.162 | **0.287** | **0.329** | **1** | **0.361** | **0.420** | **0.290** | **0.446** | **0.454** | 0.132 | **0.476** | **0.785** | **0.440** | **0.434** | **0.373** | 0.138 | **0.392** | -0.032 | **0.389** | **0.366** | **0.373** | **0.479** |
| SM18:1/22:1 | 0.220 | **0.322** | **0.420** | 0.177 | **0.489** | 0.272 | **0.417** | 0.194 | **0.817** | **0.374** | 0.217 | **0.669** | **0.884** | **0.361** | **1** | **0.669** | **0.850** | **0.822** | **0.584** | 0.055 | **0.951** | **0.403** | **0.849** | **0.695** | **0.956** | **0.745** | **0.824** | 0.222 | **0.952** | **0.953** | **0.845** | 0.198 |
| SM18:0/22:0 | **0.286** | **0.615** | **0.893** | 0.264 | **0.681** | **0.311** | **0.423** | 0.183 | **0.824** | **0.304** | 0.222 | **0.753** | **0.566** | **0.420** | **0.669** | **1** | **0.653** | **0.944** | **0.979** | -0.020 | **0.657** | **0.555** | **0.879** | **0.967** | **0.739** | **0.603** | **0.864** | 0.094 | **0.713** | **0.724** | **0.769** | 0.259 |
| SM18:1/23:1 | **0.341** | **0.331** | **0.460** | 0.231 | **0.460** | **0.332** | **0.403** | **0.296** | **0.766** | **0.417** | 0.188 | **0.795** | **0.877** | **0.290** | **0.850** | **0.653** | **1** | **0.769** | **0.563** | 0.198 | **0.864** | 0.279 | **0.764** | **0.630** | **0.847** | **0.838** | **0.714** | 0.163 | **0.823** | **0.784** | **0.678** | 0.034 |
| SM18:1/23:0 | 0.243 | **0.576** | **0.788** | 0.197 | **0.673** | **0.298** | **0.452** | 0.227 | **0.841** | **0.364** | 0.241 | **0.772** | **0.707** | **0.446** | **0.822** | **0.944** | **0.769** | **1** | **0.906** | 0.022 | **0.810** | **0.585** | **0.912** | **0.946** | **0.874** | **0.706** | **0.951** | 0.135 | **0.844** | **0.863** | **0.891** | 0.240 |
| SM18:0/23:0 | 0.244 | **0.605** | **0.931** | 0.238 | **0.659** | **0.294** | **0.394** | 0.123 | **0.763** | 0.253 | 0.205 | **0.662** | **0.459** | **0.454** | **0.584** | **0.979** | **0.563** | **0.906** | **1** | -0.046 | **0.593** | **0.627** | **0.856** | **0.960** | **0.673** | **0.533** | **0.839** | 0.050 | **0.636** | **0.659** | **0.721** | 0.259 |
| SM18:1/24:3 | **0.323** | 0.243 | -0.012 | 0.259 | 0.150 | 0.204 | 0.066 | **0.359** | 0.010 | 0.216 | 0.087 | 0.210 | 0.216 | 0.132 | 0.055 | -0.020 | 0.198 | 0.022 | -0.046 | **1** | 0.103 | -0.017 | 0.003 | -0.043 | 0.093 | 0.232 | 0.015 | **0.557** | 0.062 | -0.004 | -0.033 | -0.156 |
| SM18:1/24:2 | 0.256 | **0.344** | **0.461** | 0.253 | **0.503** | **0.341** | **0.407** | 0.219 | **0.836** | **0.456** | 0.226 | **0.662** | **0.906** | **0.476** | **0.951** | **0.657** | **0.864** | **0.810** | **0.593** | 0.103 | **1** | **0.488** | **0.833** | **0.669** | **0.952** | **0.732** | **0.798** | 0.150 | **0.927** | **0.891** | **0.782** | 0.179 |
| Hex18:1/24:0 | 0.096 | **0.445** | **0.617** | 0.038 | **0.476** | 0.250 | **0.364** | 0.229 | **0.381** | **0.481** | **0.302** | **0.377** | 0.269 | **0.785** | **0.403** | **0.555** | 0.279 | **0.585** | **0.627** | -0.017 | **0.488** | **1** | **0.536** | **0.661** | **0.420** | **0.304** | **0.645** | -0.126 | **0.389** | **0.494** | **0.608** | 0.266 |
| SM18:0/24:1 | 0.236 | **0.467** | **0.745** | 0.247 | **0.630** | **0.365** | **0.512** | 0.217 | **0.866** | 0.246 | 0.114 | **0.643** | **0.700** | **0.440** | **0.849** | **0.879** | **0.764** | **0.912** | **0.856** | 0.003 | **0.833** | **0.536** | **1** | **0.891** | **0.891** | **0.682** | **0.890** | 0.176 | **0.876** | **0.889** | **0.809** | **0.315** |
| SM18:0/24:0 | 0.158 | **0.530** | **0.855** | 0.110 | **0.631** | 0.230 | **0.421** | 0.193 | **0.752** | 0.284 | 0.251 | **0.717** | **0.550** | **0.434** | **0.695** | **0.967** | **0.630** | **0.946** | **0.960** | -0.043 | **0.669** | **0.661** | **0.891** | **1** | **0.745** | **0.636** | **0.934** | 0.078 | **0.720** | **0.785** | **0.858** | 0.234 |
| SM18:1/25:2 | 0.227 | **0.393** | **0.526** | 0.253 | **0.562** | **0.327** | **0.418** | 0.192 | **0.846** | **0.300** | 0.178 | **0.643** | **0.861** | **0.373** | **0.956** | **0.739** | **0.847** | **0.874** | **0.673** | 0.093 | **0.952** | **0.420** | **0.891** | **0.745** | **1** | **0.754** | **0.859** | 0.249 | **0.967** | **0.931** | **0.841** | 0.226 |
| SM18:1/25:1 | 0.073 | 0.162 | **0.403** | 0.001 | **0.338** | 0.175 | **0.324** | 0.264 | **0.578** | 0.270 | **0.386** | **0.817** | **0.694** | 0.138 | **0.745** | **0.603** | **0.838** | **0.706** | **0.533** | 0.232 | **0.732** | **0.304** | **0.682** | **0.636** | **0.754** | **1** | **0.740** | 0.089 | **0.732** | **0.744** | **0.700** | -0.200 |
| SM18:1/25:0 | 0.064 | **0.412** | **0.686** | 0.006 | **0.566** | 0.179 | **0.407** | 0.230 | **0.740** | **0.292** | 0.265 | **0.701** | **0.674** | **0.392** | **0.824** | **0.864** | **0.714** | **0.951** | **0.839** | 0.015 | **0.798** | **0.645** | **0.890** | **0.934** | **0.859** | **0.740** | **1** | 0.124 | **0.832** | **0.904** | **0.961** | 0.165 |
| SM18:1/26:3 | **0.301** | 0.215 | -0.013 | **0.307** | 0.209 | 0.120 | 0.014 | 0.240 | 0.133 | -0.073 | -0.105 | 0.056 | 0.229 | -0.032 | 0.222 | 0.094 | 0.163 | 0.135 | 0.050 | **0.557** | 0.150 | -0.126 | 0.176 | 0.078 | 0.249 | 0.089 | 0.124 | **1** | 0.224 | 0.198 | 0.148 | 0.140 |
| SM18:1/26:2 | 0.232 | **0.386** | **0.490** | 0.258 | **0.562** | **0.394** | **0.502** | 0.242 | **0.840** | **0.372** | 0.192 | **0.635** | **0.849** | **0.389** | **0.952** | **0.713** | **0.823** | **0.844** | **0.636** | 0.062 | **0.927** | **0.389** | **0.876** | **0.720** | **0.967** | **0.732** | **0.832** | 0.224 | **1** | **0.951** | **0.841** | **0.287** |
| SM18:1/26:1 | 0.115 | **0.339** | **0.499** | 0.088 | **0.542** | **0.292** | **0.515** | 0.247 | **0.765** | **0.317** | 0.224 | **0.640** | **0.781** | **0.366** | **0.953** | **0.724** | **0.784** | **0.863** | **0.659** | -0.004 | **0.891** | **0.494** | **0.889** | **0.785** | **0.931** | **0.744** | **0.904** | 0.198 | **0.951** | **1** | **0.929** | 0.248 |
| SM18:1/26:0 | 0.013 | **0.346** | **0.536** | -0.049 | **0.496** | 0.127 | **0.373** | 0.207 | **0.668** | **0.337** | **0.297** | **0.657** | **0.684** | **0.373** | **0.845** | **0.769** | **0.678** | **0.891** | **0.721** | -0.033 | **0.782** | **0.608** | **0.809** | **0.858** | **0.841** | **0.700** | **0.961** | 0.148 | **0.841** | **0.929** | **1** | 0.191 |
| Lac18:1/18:0 | **0.400** | **0.568** | **0.330** | **0.447** | **0.557** | **0.556** | **0.570** | 0.259 | **0.323** | 0.175 | **-0.476** | -0.125 | 0.048 | **0.479** | 0.198 | 0.259 | 0.034 | 0.240 | 0.259 | -0.156 | 0.179 | 0.266 | **0.315** | 0.234 | 0.226 | -0.200 | 0.165 | 0.140 | **0.287** | 0.248 | 0.191 | **1** |

*Values in bold are significant (P<0.05)

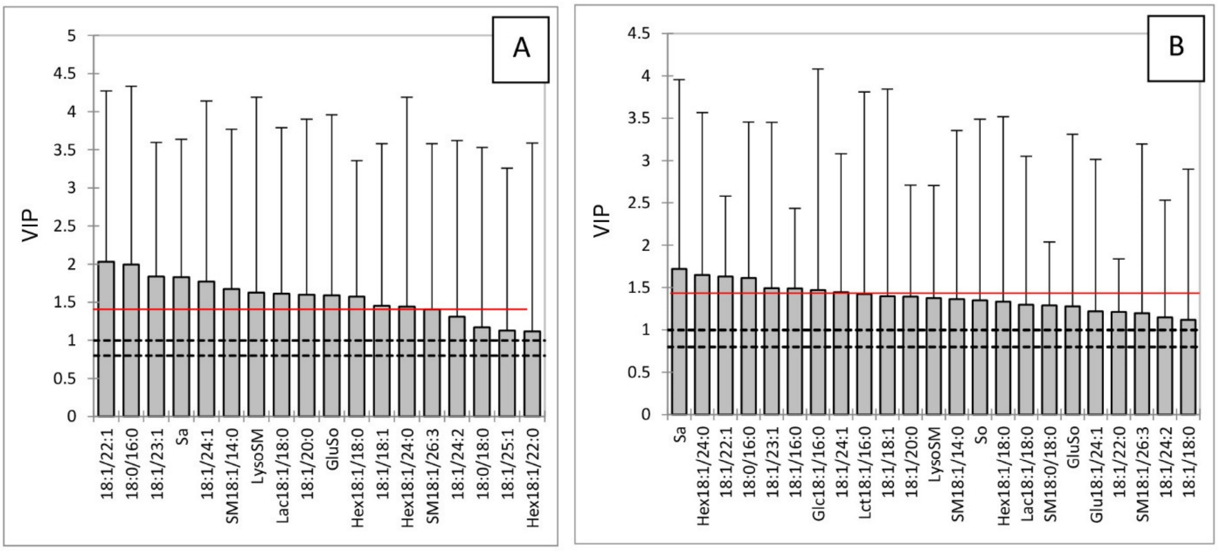


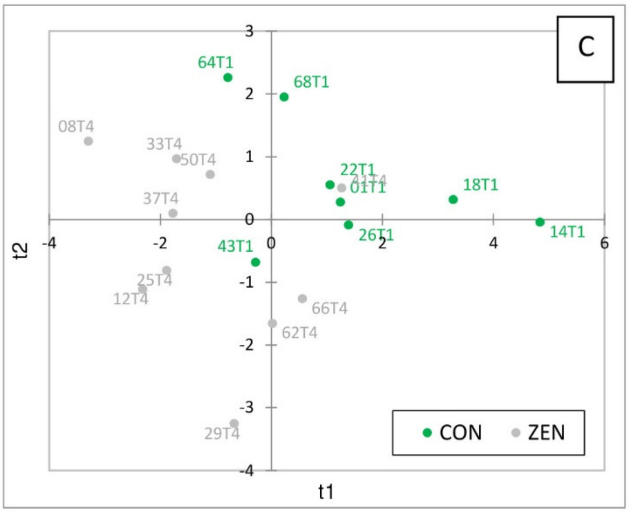


D

Model quality (VIP > 1.4):

| | Comp1 | Comp2 | Comp3 | Comp4 |
|---|---|---|---|---|
| $Q^2$ cum | 0.205 | 0.103 | 0.017 | -0.764 |
| $R^2Y$ cum | 0.369 | 0.516 | 0.572 | 0.645 |
| $R^2X$ cum | 0.264 | 0.428 | 0.634 | 0.672 |

Confusion matrix:

| From\To | CON | FB | Total | Correct (%) |
|---|---|---|---|---|
| CON | 8 | 0 | 8 | 100 |
| ZEN | 0 | 10 | 10 | 100 |
| Total | 8 | 0 | 18 | 100 |

E

Model quality (VIP > 1.1)

| | Comp1 | Comp2 | Comp3 | Comp4 |
|---|---|---|---|---|
| $Q^2$ cum | 0.108 | -0.195 | -0.344 | -1.153 |
| $R^2Y$ cum | 0.355 | 0.540 | 0.570 | 0.655 |
| $R^2X$ cum | 0.225 | 0.336 | 0.650 | 0.705 |

**Figure S1.** Partial least squares discriminant analysis (PLS-DA) of sphingolipids measured in the livers of turkeys fed a control diet (CON, T1) free of mycotoxins and a diet containing zearalenone (ZEN, T4) at a concentration of 0.47 mg/kg. Scores of the variables that are important in the projection (VIP) of the first (A) and the second (B) components. C: Discrimination of the factor axes extracted from the original explanatory variables. D: Quality of the model and confusion matrix for the training sample (variable groups) using VIP with a score > 1.4. E: Quality of the model for the training sample (variable groups) using VIP with a score > 1.1.

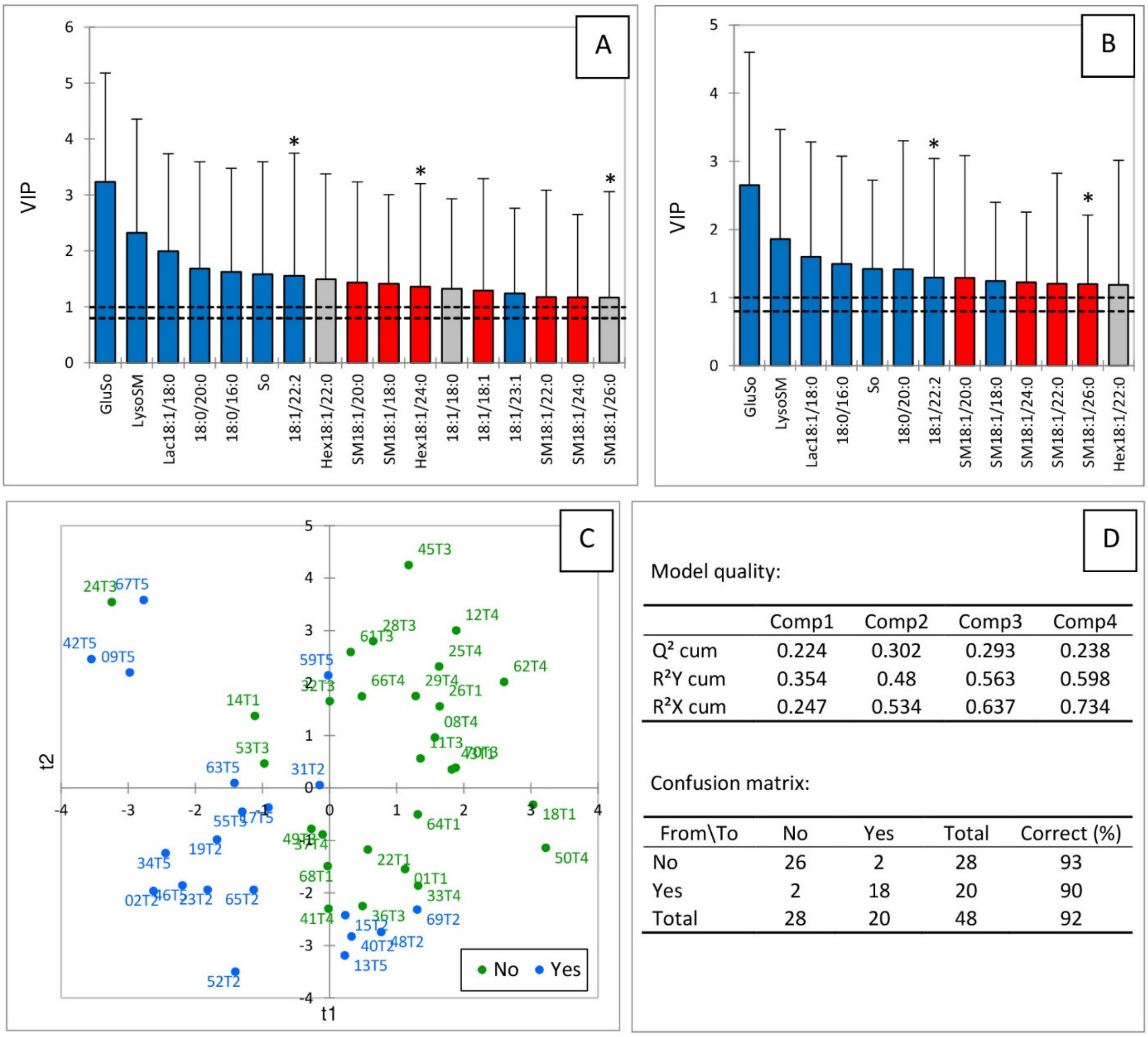


Model quality:

| | Comp1 | Comp2 | Comp3 | Comp4 |
|---|---|---|---|---|
| $Q^2$ cum | 0.224 | 0.302 | 0.293 | 0.238 |
| $R^2Y$ cum | 0.354 | 0.48 | 0.563 | 0.598 |
| $R^2X$ cum | 0.247 | 0.534 | 0.637 | 0.734 |

Confusion matrix:

| From\To | No | Yes | Total | Correct (%) |
|---|---|---|---|---|
| No | 26 | 2 | 28 | 93 |
| Yes | 2 | 18 | 20 | 90 |
| Total | 28 | 20 | 48 | 92 |

**Figure S2.** Partial least squares discriminant analysis (PLS-DA) of sphingolipids measured in the livers of turkeys fed 5 experimental diets according to the presence (Yes) or the absence (No) of deoxynivalenol in the diets. The 5 experimental diets corresponded to a control diet (CON, T1) free of mycotoxins, a diet containing fumonisins (FB, T3) at a concentration of 20.2 mg FB1+FB2/kg, a diet containing deoxynivalenol (DON, T2) at a concentration of 5.12 mg/kg, a diet containing zearalenone (ZEN, T4) at a concentration of 0.47 mg/kg, and a diet containing a combination of fumonisins, deoxynivalenol and zearalenone (FDZ, T5) at concentrations of respectively, 25.7, 5.15 and 0.57 mg/kg feed. Scores of the variables that are important in the projection (VIP) of the first (A) and the second (B) components. C: Discrimination of the factor axes extracted from the original explanatory variables. D: Quality of the model and confusion matrix for the training sample (variable groups). * 18:1/22 has a VIP score of 0.933 for the second component in PLS-DA analysis of sphingolipids obtained from chickens fed the DON and the CON diets. Hex18:1/24:0 and SM18:1/26:0 have VIP scores of 0.907 for the first component in PLS-DA analysis of sphingolipids obtained from chickens fed the FB and the CON diets.

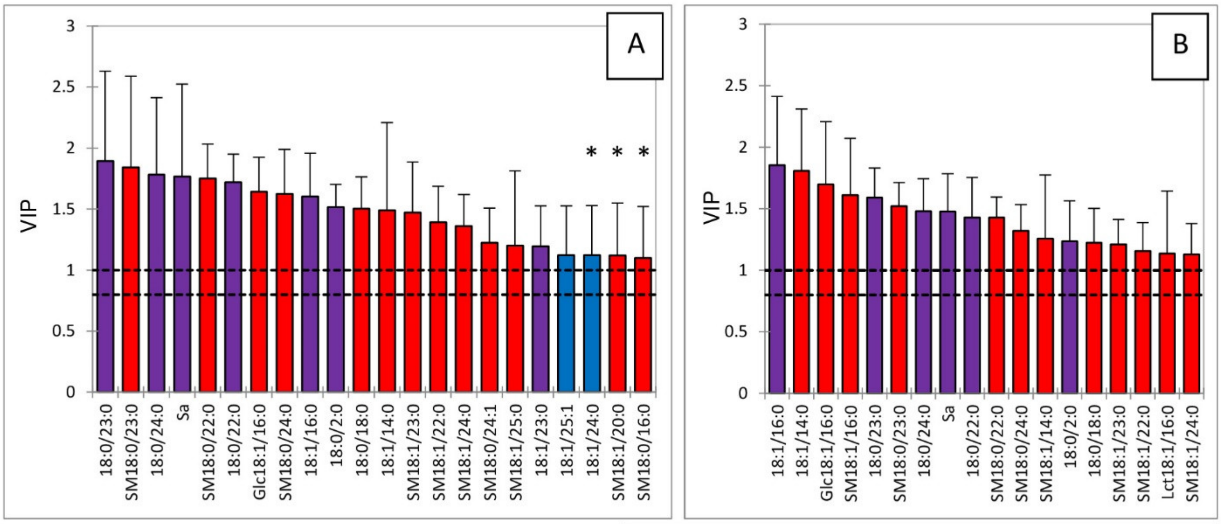


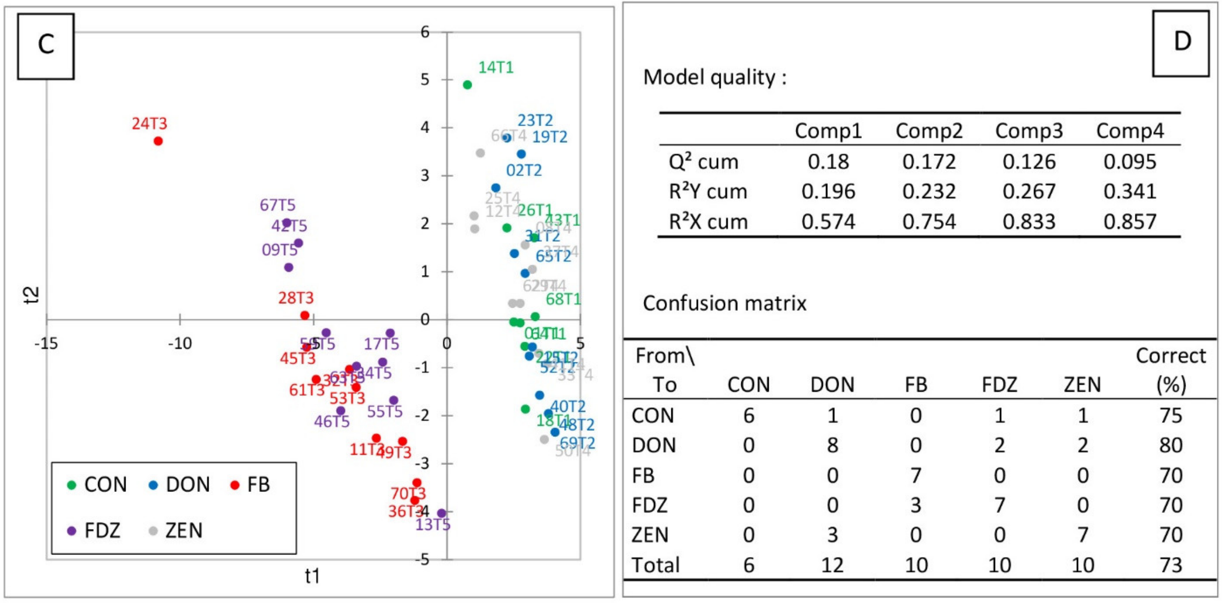


D

Model quality :

| | Comp1 | Comp2 | Comp3 | Comp4 |
|---|---|---|---|---|
| $Q^2$ cum | 0.18 | 0.172 | 0.126 | 0.095 |
| $R^2Y$ cum | 0.196 | 0.232 | 0.267 | 0.341 |
| $R^2X$ cum | 0.574 | 0.754 | 0.833 | 0.857 |

Confusion matrix

| From\ To | CON | DON | FB | FDZ | ZEN | Correct (%) |
|---|---|---|---|---|---|---|
| CON | 6 | 1 | 0 | 1 | 1 | 75 |
| DON | 0 | 8 | 0 | 2 | 2 | 80 |
| FB | 0 | 0 | 7 | 0 | 0 | 70 |
| FDZ | 0 | 0 | 3 | 7 | 0 | 70 |
| ZEN | 0 | 3 | 0 | 0 | 7 | 70 |
| Total | 6 | 12 | 10 | 10 | 10 | 73 |

**Figure S3.** Partial least squares discriminant analysis (PLS-DA) of sphingolipids measured in the livers of turkeys fed 5 experimental diets corresponding to a control diet (CON, T1) free of mycotoxins, a diet containing fumonisins (FB, T3) at a concentration of 20.2 mg FB1+FB2/kg, a diet containing deoxynivalenol (DON, T2) at a concentration of 5.12 mg/kg, a diet containing zearalenone (ZEN, T4) at a concentration of 0.47 mg/kg, and a diet containing fumonisins, deoxynivalenol and zearalenone in combination (FDZ, T5) at the respective concentrations of 25.7, 5.15 and 0.57 mg/kg feed. Scores of the variables that are important in the projection (VIP) of the first (A) and the second (B) components. C: Discrimination on the factor axes extracted from the original explanatory variables. D: Quality of the model and confusion matrix of the training sample (variable groups). * 18:1/24:0 has a VIP score of 0.99 for the second component in PLS-DA analysis of sphingolipids obtained from chickens fed the DON and the CON diets. SM18:0/16:0 and SM18:1/20:0 have VIP scores of 1.037 and 1.068, respectively, for the first component in PLS-DA analysis of sphingolipids obtained from chickens fed the FB and the CON diets.
